# Inter- and intrarater reliability of Minimal Eating Observation and Nutrition Form – version II (MEONF-II) nurse assessments among hospital inpatients

**DOI:** 10.1186/1472-6955-13-18

**Published:** 2014-07-08

**Authors:** Albert Westergren, Ólina Torfadóttir, Peter Hagell

**Affiliations:** 1 The PRO-CARE Group, School of Health and Society, Kristianstad University, Kristianstad SE-291 88, Sweden; 2 Akureyri University Hospital - Regional Hospital, Akureyri, Iceland

**Keywords:** Undernutrition, MEONF-II, Interrater reliability, Intrarater reliability

## Abstract

**Background:**

The Minimal Eating Observation and Nutrition form – version II (MEONF – II) is a recently developed nursing nutritional screening tool. However, its inter- and intrarater reliability has not been assessed.

**Methods:**

Inpatients (n = 24; median age, 69 years; 11 women) were assessed by eight nurses (interrater reliability, two nurses scored each patient independently) using the MEONF-II on two consecutive days (intrarater reliability, each patient was scored by the same nurse day 1 and day 2).

**Results:**

Six patients were at moderate/high undernutrition risk. Inter- and intrarater reliabilities (Gwet’s agreement coefficient) for the MEONF-II 2-category classification (no/low risk versus moderate/high risk) were 0.93 and 0.81; for the 3-category classification (no/low – moderate – high risk) reliabilities (Gwet’s weighted agreement coefficient) were 0.98 and 0.88; and total score inter- and intrarater reliabilities (intraclass correlation) were 0.92 and 0.84.

**Conclusion:**

Reliability of MEONF-II nurse assessments among adult hospital inpatients was supported and the tool can be used in research and clinical practice.

## Background

The Minimal Eating Observation and Nutrition form version II (MEONF-II) [1-3] was developed to be used by nurses as it is these that most often conduct the initial nutritional screening. Studies have supported the validity of the MEONF-II [1-3], and its user-friendliness is high among registered nurses [1,2] and among nursing students [4]. Although interrater reliability among hospital nurses’ assessments according to its predecessor (the MEONF-I) has been supported (weighted Kappa, 0.81) [5], inter- and intrarater reliability of assessments using the MEONF-II remains to be documented.

The MEONF-II (Additional file 1) is based on recommendations for detecting undernutrition risk [6-8] and includes assessments of unintentional weight loss, low BMI/short calf circumference, and eating difficulties. The included eating difficulties (food intake, chewing/swallowing, energy/appetite) are based on the Minimal Eating Observation Form – Version II (MEOF-II) [9,10]. An additional assessment of the presence or absence of clinical signs of undernutrition is also included [2]. MEONF-II scores range from 0–8 (0–2 = low risk; 3–4 = moderate risk; ≥5 = high risk for undernutrition) [3]. MEONF-II has shown acceptable sensitivity (0.61-0.73), specificity (0.79-0.88) and accuracy (0.68-0.82) compared to the Mini Nutritional Assessment (MNA, 18 item version) [1-3]. The aim of this study was to evaluate the inter- and intrarater reliability of the MEONF-II assessments among adult inpatients.

## Methods

Data were collected at a regional Icelandic hospital [11] using a cross-sectional test-retest design. The Guidelines for Reporting Reliability and Agreement Studies (GRRAS) were followed [12].

### Participants

Adult inpatients (≥18 years old) in four general adult hospital wards (surgery, general medicine, rehabilitation for young and old people) were eligible for inclusion. Fifty-six (76%) of 74 available inpatients gave informed consent to participate, of which every second (n = 28) was included in the study of inter- and intrarater reliability. The other patients were included for the purpose of estimating the pointprevalence of undernutrition risk at the hospital (not presented here). Four patients were not available for intrarater reliability assessment on day two and were thus excluded. The final sample was 24 patients. For test-retest reliability studies a minimal sample size of 15 to 20 suffice [13,14].

### Data collection

Two registered nurses at each of the four participating wards (n = 8) performed the data collection. Before commencing data collection, they received brief training (45–60 minutes), including pre-testing of the forms.

Data were collected in 2012. Two nurses on each ward assessed the patients according to the MEONF-II, in parallel with each other (day 1, interrater reliability). The nurses did not communicate during the assessment or about their findings afterwards. The nurses in each pair agreed upon who should be designated to be the "first" and "second nurse" (for the purpose of interrater reliability). During day 2 the MEONF-II assessment was repeated, by the same nurse who made the assessment of the same patient on day 1, to assess the intrarater reliability.

MEONF-II height and weight measurements were conducted using standard equipment available at the included units, and the patients were observed while eating and asked about eating difficulties and unintentional weight loss. Data collection was made under conditions as close as possible to clinical daily routine.

### Instruments

The data collection forms, including the MEONF-II and the manual were translated into Icelandic and back translated into Swedish in collaboration with a professional translator.

In addition to the MEONF-II, dependence in activities of daily living (ADL) was assessed using a modified Katz ADL-index [4,15]. It summarises an individual’s overall performance in six activities: hygiene; dressing and undressing; ability to go to the toilet; mobility; ability to control bowels and bladder; and food intake. Patients were then classified as almost totally dependent (help in 5–6 activities), partly dependent (help in 3–4 activities), or almost totally independent (help in 2 activities or less) [4].

In addition, three single-items regarding fatigue/tiredness, depression and perceived health were applied. The fatigue/tiredness and depression items asked whether respondents had gotten tired without any specific reason and felt gloomy and depressed, respectively, today/during the last days (graded as: not at all; yes, a little; yes, quite a lot; a lot) [4,16,17]. The perceived health item asked how respondents perceived their health in comparison with other people of the same age (grades as: not as good as others; as good as others; better than others) [4,18].

### Analysis

Comparisons were made between those included in the study and those not using Chi-square and Mann–Whitney *U* test depending on level of data. Inter- and intrarater reliability was analysed with proportion exact agreement (PA), Kappa statistics (*K*), quadratic weighted *K* (*K*_*w*_), Gwet’s agreement coefficient (AC1), weighted AC1 (AC1_*w*_), and intraclass correlation coefficient (ICC) and their 95% Confidence Intervals (95% CIs) [19-22]. Since the MEONF-II generates different outcomes, these statistics were primarily interpreted in association with the following outcomes: *K* and AC1 for the 2-category classification (identifying no/low risk versus moderate/high risk), *K*_w_ and AC1_*w*_ for the 3-category classification (no/low – moderate – high risk), and ICC for the total score [23].

PA does not account for chance agreement but is useful as a complement to other statistics, particularly to *K* when there are low frequencies in some cells. The advantage of *K* is that it accounts for both PA and the chance agreement, and *K*_*w*_ considers partial agreement [24]. However, *K* statistics can sometimes be low despite high levels of agreement [23]. In order to facilitate the interpretation of *K* we calculated the maximum obtainable kappa (*K*_max_), and the prevalence-adjusted bias-adjusted kappa (PABAK). If the prevalence index is high, i.e. the prevalence of a positive rating is very high or low, chance agreement is also high and *K* is reduced accordingly. A bias index close to zero indicates that the disagreement between raters is close to symmetrical. The higher the bias index is, the higher the *K* will be. Thus, PABAK adjusts for both prevalence and bias. *K*_max_ shows the maximum obtainable kappa for the set of data used [23]. The AC1 statistics is more robust than *K* statistics and has therefore been recommended as an alternative or complement to *K*[25]. *K* and AC1 values below 0.20 are regarded as poor, 0.21-0.40 as fair, 0.41-0.60 as moderate, 0.61-0.80 as substantial and >0.80 as almost perfect agreement [25,26].

Regarding the ICC we used a two-way mixed model (subjects random, ratings fixed, single measurement, absolute agreement) [27]. ICC values should preferably exceed 0.8 but values above 0.70 are considered acceptable [24]. Data were analysed using IBM SPSS Statistics 20, MedCalc 12 and AgreeStat 2011.1 for Windows.

### Ethical considerations

The ethical committee of Akureyri Hospital (case 2/2012) approved the study and the use of personal data from the study was notified to the Data Protection Authority (S5594/2012). The study conforms to the provisions of the Declaration of Helsinki in 1995 (as revised in Edinburg 2000) including informed consent and patient anonymity [28].

## Results

The eight nurses (all females) had a median age of 51 years (min-max, 40–60 years), had been working as nurses for a median of 16 years (7–38 years), and in the current hospital for a median of 12 years (4–32 years). The nurses were general hospital ward nurses and none of them had prior experience with the MEONF-II.

There were no significant differences between patients included in the study and those not (Table 1).

**Table 1:** Background data for the sample (n = 24) and comparisons with those not included (n = 32) regarding age, gender and type of ward

	**Included, n = 24**	**Not included, n = 32**	**P-value**
**Age**			0.177^a)^
Median (min-max)	69 (33–92)	69 (22–94)	
	**n (%)**	**n (%)**	
**Gender**, women	11 (46)	18 (56)	0.440^b)^
**Type of ward**			0.710^b)^
Medical	4 (17)	8 (25)	
Surgical	9 (37)	14 (44)	
Rehabilitation, young	4 (17)	4 (12)	
Rehabilitation, older	7 (29)	6 (19)	
**Common diagnose categories (can have more than one)**			
Lung	5 (22)		
Cardiovascular	9 (39)		
Gastrointestinal	5 (22)		
Orthopaedic	8 (35)		
**ADL categories**			
Almost totally independent (max 2 activities)	12 (50)		
Partly dependent (3–4 activities)	6 (25)		
Almost totally dependent (5–6 activities)	6 (25)		
**Health compared to others**			
Not as good as	14 (58)		
As good as	10 (42)		
Better than others	0		
**Tired without reason**			
Not at all	9 (37)		
Yes, a little	8 (33)		
Yes, quite a lot	3 (12)		
Yes, a lot	4 (17)		
**Low-spiritedness**			
Not at all	13 (54)		
Yes, a little	6 (25)		
Yes, quite a lot	4 (17)		
Yes, a lot	1 (4)		

^a)^Mann–Whitney *U* test.

^b)^Chi-square test.

The prevalence of moderate/high undernutrition risk on day 1 was 21-25%. On day 2 the prevalence was 21% (Table 2).

**Table 2:** Descriptive nutritional risk screening data according to the Minimal Eating Observation and Nutrition Form (MEONF-II)

	**"First" nurse assessments, day 1**^**a)**^	**"Second" nurse assessments, day 1**^**a)**^	**Nurse assessments, day 2**^**a)**^
Unintentional weight loss, n (%)	4 (17)	4 (17)	5 (21)
Low Body Mass Index or short Calf Circumference, n (%)	1 (4)	2 (8)	1 (4)
**Problem with…**			
…food intake, n (%)^b)^	6 (25)	7 (29)	6 (25)
…swallowing/mouth, n (%)^c)^	4 (17)	2 (8)	8 (33)
…energy/appetite, n (%)^d)^	6 (25)	4 (17)	6 (25)
Clinical signs indicate risk of undernutrition, n (%)	5 (21)	4 (17)	4 (17)
**MEONF-II total score**^e)^			
Mean (SD)	1.5 (1.8)	1.2 (1.5)	1.6 (1.8)
Median (q1-q3)	1.0 (0–3)	0.5 (0–2)	1.5 (0–2)
Minimum-Maximum	0–6	0–5	0–6
**MEONF-II risk category**			
No/Low risk, n (%)	18 (75)	19 (79)	19 (79)
Moderate risk, n (%)	5 (21)	4 (17)	2 (8)
High risk, n (%)	1 (4)	1 (4)	3 (13)

^a)^The "first" (n = 4) and "second" nurses (n = 4) conducted parallel but independent assessments of patients on day 1 and repeated their assessments on day 2 (the same nurses for the same patients as on day 1).

^b)^Sitting position; Manipulating food on the plate; Conveying food to the mouth.

^c)^Chewing; Coping with food in the mouth; Swallowing.

^d)^Eats less than ¾ of served food; Lacks energy to complete an entire meal; Poor appetite.

^e)^Possible score range, 0–8 (8 = worst).

The inter- and intrarater reliabilities (PA/*K*/AC1) for the MEONF-II 2-category classification (no/low risk versus moderate/high risk) were ≥0.81 except for intrarater *K* (0.65). Similarly, for the 3-category classification (no/low – moderate – high risk) inter- and intrarater reliabilities (PA/*K*_w_/AC1_w_) were were ≥0.83 except for intrarater *K*_w_ (0.62). For the total MEONF-II scores ICC values were above 0.80 for both inter- and intrarater reliabilities (Table 3).

**Table 3:** Inter- and intrarater reliability of MEONF-II scores (n = 24) as assessed by eight nurses

	**PA (95% CI)**	***K *** **(95% CI)**	**AC1**	***K***_***w ***_**(95% CI)**	**AC1**_***w***_	**ICC (95% CI)**^**g)**^
**Interrater-reliability**						
2 categories^a)^	0.96 (0.87-0.1.0)	**0.88 (0.65-1.0)**^**c)**^	**0.93 (0.80-1.0)**	NA	NA	NA
3 categories^b)^	0.96 (0.87-1.0)	0.89 (0.66-1.0)^d)^	NA	**0.93 (0.76-1.0)**^**d)**^	**0.98 (0.95-1.0)**	0.93 (0.84-0.97)
Total score	0.67 (0.47-0.87)	0.53 (0.31-0.75)	NA	0.91 (0.84-0.99)^c)^	**0.98 (0.96-1.0)**	**0.92 (0.80-0.96)**
**Intrarater-reliability**						
2 categories^a)^	0.88 (0.74-1.0)	**0.65 (0.26-1.0)**^**e)**^	**0.81 (0.57-1.0)**	NA	NA	NA
3 categories^b)^	0.83 (0.68-0.99)	0.57 (0.21-0.92)^f)^	NA	**0.62 (0.19-1.0)**^**f)**^	**0.88 (0.72-1.0)**	0.63 (0.30-0.82)
Total score	0.67 (0.47-0.87)	0.56 (0.33-0.78)	NA	0.82 (0.66-0.98)	**0.95 (0.91-.1.0)**	**0.84 (0.67-0.93)**

^a)^2-category classification = no/low vs moderate/high risk.

^b)^3-category classification = no/low vs moderate vs high risk.

^c)^*K*_max_ = 0.88, Prevalence-adjusted bias-adjusted kappa (PABAK) = 0.92.

^d)^*K*_max_ (weighted or unweighted) = 0.89, PABAK = 0.98.

^e)^*K*_max_ = 0.88, PABAK = 0.75.

^f)^*K*_max_ (weighted or unweighted) = 0.89, PABAK = 0.85.

^g)^Two-way mixed model for single measures, absolute agreement criterion.

PA, Proportion exact agreement; *K,* Kappa; *K*_*w*_*,* weighted Kappa (quadratic weights); ICC, Intraclass Correlation Coefficient; AC1, Gwet’s first order agreement coefficient. AC1_*w*_, Gwet’s first order agreement coefficient (quadratic weights); NA, not applicable

Data in boldface indicate the most appropriate statistic in relation to the respective MEONF-II outcomes.

## Discussion

This study addressed the inter- and intrarater reliability of MEONF-II nurse assessments of adult hospital inpatients. Our observations suggest that such assessments meet generally acceptable criteria for inter- and intrarater reliability.

The sample does not appear to differ from other hospital samples, as we found an undernutrition rate (moderate/high risk) of 21 or 25%. This is similar to previously reported rates according to the MEONF-II. In an Icelandic study [11] the baseline prevalence of undernutrition (moderate/high risk) was 25%, and in Swedish small, middle and large sized hospitals the corresponding rates have been 22-34% [29]. This implies that, from a nutritional perspective, the sample in this study seems to be a representative hospital sample. Furthermore, there are no apparent reasons to question the representativeness of the participating nurses.

MEONF-II assessments were found to exhibit generally good reliabilities following a relatively low amount of training. The interrater reliability was particularly high and comparable to the figures found for other nutritional screening tools. The interrater reliability for MEONF-II total score (*K* 0.53) is similar to that found for the Mini Nutritional Assessment total score (MNA, *K* 0.51) between two geriatric clinicians independently assessing 39 in-hospital patients [30]. Furthermore, a review of the Subjective Global Assessment (SGA) found that *K* varied between 0.34 and 0.88, and was highest with experienced assessors [31]. In another study of the MNA in long-term geriatric clinics (two nurses with more than one year’s experience from using the MNA, median time between assessments 12 days), intrarater reliability was 0.89 (ICC) for total MNA scores and 0.78 (*K*_*w*_) for the 3-category classifications [32]. These somewhat better intrarrater reliabilities compared to those observed here may be explained by differences in settings (i.e., hospital inpatients might be less stable than residents in long term care facilities). However, in line with the observations by Steenson et al. [31] it appears more likely to be related to the assessors’ long experience with the tested tool. Additional studies are needed to examine the effect of training on the reliability of MEONF-II scores.

Arguably, the most important reliability is that of the 2-category classification of MEONF-II scores as it initially might be used for determining if a patient shall receive a nutritional intervention and/or a more thorough assessment. This reliability was found to be high (AC1, 0.93) between raters but lower (albeit still acceptable) within raters (AC1, 0.81). This may be due to several possible explanations. There might have been a change in nutritional risk between the two time points, due to improvement or decline in health status. Differences in total scores in assessments between the 24 hours affected the nutritional risk classifications of four patients; two in each direction, i.e. towards better and worse nutritional risk status, respectively. The choice of time period between assessments is primarily a balance between the risk for changes in the underlying trait and recall effects [27]. A second explanation that also is supported in our data is that it relates to the observed score distributions. When there is a low bias, kappa is lower than when bias is large, and when prevalence is very high or low the chance agreement increase and reduces *K*[23], whereas the AC1 statistic appears more robust in such situations [25]. The difference between *K* and PABAK provides some support for this explanation. However, the strongest support is seen in the differences between *K* (0.65), AC1 (0.81) and PA (0.88). Indeed, when *K* is low despite high PA it has been suggested to instead rely on the more robust AC1 [25]. Our observations provide additional support to that view. However, the fact remains that the reliability, even according to AC1, between raters was higher than within raters, which therefore most likely appears related to actual changes in nutritional risk between the two time points.

According to Kottner et al. (12), reliability values of 0.60-0.80 may be regarded as sufficient for group-level applications, whereas values of at least 0.90 are required for individual patient assessments, when important decisions are based on the assessment [12]. Thus, from this perspective the MEONF-II performs well at both a group and (in most instances) individual level and can be used for both for research purposes as well as in clinical practice.

## Conclusions

This study provides support for the inter- and intrarater reliability of MEONF-II nurse assessments among adult hospital inpatients. Somewhat compromised intrarater reliability according to *K* statistics, despite high proportions of exact agreement, is likely to represent a methodological artefact. The MEONF-II can be used in a reliable way in research and clinical practice.

## Competing interests

The authors declare that they have no competing interests.

## Authors’ contributions

All three authors have substantially contributed to the study. AW conceived and designed the study, carried out the analyses and drafted the manuscript. ÓT coordinated the translation process and the data collection. PH and ÓT revised the manuscript and interpreted the data. All three authors gave final approval of the version to be published.

## Pre-publication history

The pre-publication history for this paper can be accessed here:

http://www.biomedcentral.com/1472-6955/13/18/prepub

## Supplementary Material

Additional file 1 MEONF-II (Minimal Eating Observation and Nutrition Form – Version II). Click here for file
