# Supplementary material for: Inter- and intrarater reliability of Minimal Eating Observation and Nutrition Form – version II (MEONF-II) nurse assessments among hospital inpatients
Source: BMC Nurs. 2014 Jul 8;13:18. doi: 10.1186/1472-6955-13-18 (PMC4120003; doi:10.1186/1472-6955-13-18)
Supplement: Additional file 1 — MEONF-II (Minimal Eating Observation and Nutrition Form – Version II). [file 1472-6955-13-18-S1.pdf]

MEONF-II (Minimal Eating Observation and Nutrition Form – Version II)

	**Please tick the appropriate boxes on the left and score according to instructions**		**SCORE**
1	**Unintentional weight loss** (regardless of amount of loss and of whether recent or occurred over time)	Weight loss = 2<br>No weight loss = 0<br>Don't know = 2	
2a<br><br><br><br>2b	☐ **BMI** is less than 20 (69 years or younger)<br>☐ **BMI** is less than 22 (70 years or older)<br>***If height/weight cannot be obtained, measure calf circumference (2b)***<br>☐ **Calf circumference** is less than 31 centimetres	BMI = weight (kg)/height squared (m^2)<br><br>Low BMI or small calf circumference = 1<br>Otherwise = 0	
3	**Eating difficulties**<br>Food intake<br>☐ Difficulty maintaining good sitting position during meals<br>☐ Difficulty manipulating food on plate<br>☐ Difficulty conveying food to mouth	One/more difficulties = 1<br>No difficulty = 0	
4	Swallowing/mouth<br>☐ Difficulty chewing<br>☐ Difficulty coping with food in mouth<br>☐ Difficulty swallowing	One/more difficulties = 1<br>No difficulty = 0	
5	Energy/appetite<br>☐ Eats less than ¾ of food served<br>☐ Lacks energy to complete an entire meal<br>☐ Poor appetite	One/more problems = 2<br>No problems = 0	
6	**Clinical signs** indicate risk of undernutrition. Assess e.g., body shape, subcutaneous fat, muscle mass, grip strength, oedema (fluid retention), blood tests (e.g. serum albumin).	Clinical signs indicate risk = 1<br>Otherwise = 0	
	**Sum observations 1-6 into a total score** (min = 0, max = 8)		**TOTAL SCORE:**

RISK OF UNDERNUTRITION

☐ 0-2 points = no or low risk ☐ 3-4 points = moderate risk ☐ 5 points or more = high risk

**BMI INTERPRETATION**	*69 years or younger:*	*70 years or older:*
☐ Underweight	BMI <20	BMI <22
☐ Normal	BMI 20-24.9	BMI 22-26.9
☐ Overweight	BMI 25-29.9	BMI 27-31.9
☐ Obesity	BMI 30-39.9	BMI 32-41.9
☐ Severe/morbid obesity	BMI >40	BMI >42

Comments:

© Westergren A & Hagell P. All rights reserved. No part of MEONF-II may be reproduced in any form or by electronic or mechanical means, including information storage and retrieval systems without permission in writing from Westergren A. UK-version 2014-05-06.

0-2 points
No or Low risk

Reassess:

- Hospital – once/week
- Long-term care facilities – every 3 months
- Home care – annually

3-4 points
Moderate risk

- Document fluid/dietary intake for 2-3 days
- Give nutritional drink or equivalent, possibly energy diet.
- Interventions for eating difficulties (see below)
- If improvement or adequate intake: no cause for concern; If no improvement: cause for concern – follow local policy and/or refer to dietician

5 points or more
High risk

- Referral to dietician, nutrition team and follow local policy
- Improve nutritional intake through e.g. fortified food, oral nutritional supplements (consult dietician)
- Interventions for eating difficulties (see below)
- Follow up, update care plan

Reassess & update care plan
Hospital – once/week and at discharge
Long term care facilities – at least monthly
Home care – at least every 2-3 months

All risk categories:
- Treat underlying condition and provide help and advice about food choices, eating and drinking when needed.
- Document risk category (No or low/Moderate/High risk)
- Document dietary needs and follow local guidelines

**Main steps in eating process**	***Specific interventions***<br>*Linked to main steps in eating process*	***General interventions***<br>*Linked to eating process*
**Food intake**<br>• Maintaining good sitting position during meals<br>• Manipulating food on plate<br>• Conveying food to mouth	Adapt cutlery, glass, mug.<br>Consult physiotherapist, occupational therapist.	Assistance.<br><br>Feeding.<br><br>Training.<br><br>Artificial nutrition.<br><br>Adapt mealtime environment (e.g., create a calm environment).<br><br>Reduce distractions.<br><br>Information.
**Swallowing/mouth**<br>• Chewing<br>• Coping with food in mouth<br>• Swallowing	Adapt consistency.<br>Specific swallowing techniques and head position.<br>Consult dysphagia expertise (usually speech therapist), dietician, dental hygienist/dentist.	
**Energy/appetite**<br>• Eats less than ¾ of food served<br>• Lacks energy to complete an entire meal<br>• Poor appetite	Dietary supplement.<br>Fortified food.<br>Plan other activities to preserve energy for eating.<br>Eat smaller quantities of food several times a day.<br>Consult dietician.	

Obesity
Document overweight/obesity. Check underlying reasons before initiating therapy. Refer to dietician.

© Westergren A & Hagell P. All rights reserved. No part of MEONF-II may be reproduced in any form or by electronic or mechanical means, including information storage and retrieval systems without permission in writing from Westergren A. UK-version 2014-05-06.

The MEONF-II Manual
Assessment of nutritional status: The patient/resident should be weighed and measured, preferably first thing in the morning, before breakfast. The patient/resident should only be wearing light clothing. If this is not possible, information about height and weight can be obtained from the patient chart or by asking the person. Information about weight should be recorded at least weekly (in hospital) or monthly (in long-term care).

1. Unintentional weight loss (regardless of amount of loss and of whether recent or occurred over time). Ask the patient and also review documentation relating to weight history. Ask whether rings, watch, or clothes are beginning to fit loosely. Also ask whether the person is intentionally trying to lose weight.

2a. BMI is calculated according to the following formula: weight (kg)/height squared (m^2). If height and/or weight cannot be obtained to calculate BMI – measure calf circumference instead.
2b. Calf circumference is measured in centimeters. Measure the calf at the widest point. Also measure above and below the widest point, to ensure that the first measurement was the largest.

Eating difficulties (items 3-5 below): The patient/resident should preferably be assessed by observing the individual during a meal. If this is not possible the assessment may be carried out by interviewing the patient/resident. If a person has assistive devices/assistance to be able to eat, note that the person has special needs (under "Comments"). For example, if the person has a soft diet due to swallowing difficulties, state that swallowing difficulties are present, even if they are not evident since the consistency of the diet was modified.

3. Food intake

Maintaining good sitting position during meals	Sits independently and with unrestricted mobility
Manipulating food on plate	Uses both hands, only spills occasionally, does not have plate with inner lip or special cutlery, uses traditional flatware (not a spoon for meat and potatoes), cuts food and butters bread, puts down glass unassisted
Conveying food to mouth	Coordinates arms/trunk/head when food is conveyed to mouth, does not need a bib, finds mouth without problems, only spills occasionally, no adapted equipment such as mug or straw

4. Swallowing/mouth

Chewing	Both "up and down" and rotating/grinding chewing movements. No modification of food consistency. Able to bite off pieces of food, does not rip them off. Food does not fall from mouth while chewing.
Coping with food in mouth	Food is moved to back of mouth without problems. No food remains in mouth after meals. Able to talk between bites.
Swallowing	No coughing during meals that may be attributed to aspiration. Smooth swallowing movement when food is completely chewed. No delay or concentrated effort before swallowing. Mouth essentially empty after swallowing.

5. Energy/appetite

Eats less than ¾ of food served	It is assumed that portion size is adapted to individual needs (quantity and content). Patient does not have feeding tube/IV due to inadequate food intake.
Lacks energy to complete an entire meal	Meal is interrupted due to lack of energy to continue (not due to satiety).
Poor appetite	If possible, ask directly; if not, make an assessment. Appetite should be compared with the person's usual appetite.

6. Clinical signs. Indicate risk of undernutrition. Assess e.g. body shape, subcutaneous fat, muscle mass, grip strength, oedema (fluid retention), blood tests (e.g. serum albumin)

Total score. Note that unintentional weight loss gives 2 points, as do problems related to Energy/appetite, as it is known that they are strongly associated with undernutrition.

Interpretation of MEONF-II total scores
0-2 points = No or Low risk of undernutrition
3-4 points = Moderate risk of undernutrition
5 points or more = High risk of undernutrition
When the initial assessment is carried out, proceed by planning interventions!

High BMI (overweight/obesity) is not part of the assessment of risk for undernutrition. Please note, however, that overweight/obesity may occur in the presence of undernutrition that requires intervention.

© Westergren A & Hagell P. All rights reserved. No part of MEONF-II may be reproduced in any form or by electronic or mechanical means, including information storage and retrieval systems without permission in writing from Westergren A. UK-version 2014-05-06.
